# Donepezil Plus Solifenacin (CPC-201) Treatment for Alzheimer’s Disease

**DOI:** 10.1007/s13311-016-0511-x

**Published:** 2017-01-30

**Authors:** Thomas N. Chase, Martin R. Farlow, Kathleen Clarence-Smith

**Affiliations:** 1 Chase Pharmaceuticals, Inc, 1825 K Street NW, Washington, DC 20006 USA; 2 Department of Neurology, Indiana University School of Medicine, 541 Clinical Drive, CL299, Indianapolis, IN 46202 USA

**Keywords:** Dementia, clinical trial, donepezil, solifenacin, cholinesterase inhibitor, anticholinergic.

## Abstract

Available cholinergic drugs for treating Alzheimer’s disease (AD) provide modest symptomatic benefit. We hypothesized that co-administration of a peripheral anticholinergic to reduce dose-limiting adverse effects (AEs) would enable the safe/tolerable use of higher cholinesterase inhibitor doses and thus improve their antidementia efficacy. A modified single-blind, ascending-dose, phase IIa study of donepezil plus solifenacin (CPC-201) lasting 26 weeks was conducted in 41 patients with probable AD of moderate severity. Entry criteria included the use of donepezil at a dose of 10 mg/day during the preceding 3 months. The primary outcome measure was the maximum tolerated dose (MTD) of donepezil achieved (to protocol limit of 40 mg/day) when administered with the anticholinergic solifenacin 15 mg/day. Secondary measures included assessments of cognitive and global function, as well as of AEs. The mean ± SD donepezil MTD increased to 38 ± 0.74 mg/day (median 40 mg/day; *p* < 0.001); 88% of the study population safely attained this dose at the end of titration. Markedly reduced donepezil AE frequency, especially gastrointestinal, allowed this dose increase. There were no drug-related serious AEs or clinically significant laboratory abnormalities. At 26 weeks, Alzheimer’s Disease Assessment Scale Cognitive Component scores in the efficacy evaluable population improved by 0.35 ± 0.85 points over baseline (*p* < 0.05), an estimated 2.5 ± 0.84 points above 10 mg/day donepezil and 5.4 ± 0.84 points above historic placebo (both *p* < 0.05). Clinical Global Impression of Improvement scores improved by 0.94 ± 0.20 to 3.1 ± 0.20 points (*p* < 0.001). The findings suggest that limiting donepezil AEs by co-administration of solifenacin allows the safe administration of substantially higher cholinesterase inhibitors doses that may augment cognitive and global benefits in patients with AD.

**Electronic supplementary material:**

The online version of this article (doi:10.1007/s13311-016-0511-x) contains supplementary material, which is available to authorized users.

## Introduction

### Background

Alzheimer’s disease (AD) is a progressive neurodegenerative disorder afflicting mainly the elderly [1]. Its cause remains obscure and no treatment has yet been shown to prevent onset or delay progression. Since clinical decline is associated with a profound loss of cholinergic neurons arising in the medial forebrain nuclei and an associated reduction in acetylcholine-mediated neurotransmission [2], drugs tending to normalize acetylcholine transmitter levels, such as donepezil and related cholinesterase inhibitors (ChEIs), have for over 2 decades served as the mainstay of symptomatic therapy [3]. Unfortunately, none has proven more that modestly effective: even at maximum tolerated doses (MTD), most patients with Alzheimer’s disease (AD) achieve only marginal clinical benefits [4, 5].

A major factor contributing to this limited efficacy is the marginal 25% to 30% inhibition of cortical ChE activity achieved with standard of care 5 or 10 mg/day donepezil doses [6, 7]. Higher dosing can achieve greater levels of ChE inhibition [8], but dosing is limited in most patients by adverse events (AEs), especially nausea, vomiting, and diarrhea [9]. Indeed, a 23-mg donepezil formulation, introduced in 2010 [10], gained relatively limited acceptance due mainly to gastrointestinal (GI) intolerance [11]. This is regrettable as both animal model [12–14] and AD patient [10, 15–18] studies suggest that, over a broad range, larger ChEI doses potentially confer greater cognitive benefit.

The foregoing observations led us to hypothesize that ChEI doses well above those currently approved, but rendered safe and tolerable by the co-administration of a second drug capable of inhibiting peripherally mediated cholinergic AEs, might achieve substantially greater symptomatic benefit and cognitive stabilization to those suffering from AD. Initial support for this view derived from multiple phase I studies showing that the addition of a peripheral anticholinergic to donepezil or other ChEI allows substantial increases in the MTD and associated plasma concentrations [19]. This first-in-patient study was thus designed to evaluate whether the MTD of a ChEI could be substantially raised in patients with AD by the co-administration of a peripheral anticholinergic, and, secondarily, whether this additional agent might improve cognitive function. The ChEI donepezil and the antimuscarinic solifenacin were selected as an optimal drug combination to test this hypothesis.

Donepezil hydrochloride is a selective, reversible ChEI known to enhance cholinergic function both centrally and peripherally [20]. Initially approved for US marketing in 1996 for the treatment of AD, it has been sold generically since 2010 at daily doses of 5, 10, and, most recently, 23 mg. Peak plasma concentrations of donepezil, which is 100% orally bioavailable, are achieved in approximately 3 h and the biological half-life is about 70 h.

Solifenacin is a competitive cholinergic receptor antagonist, relatively selective for the M3 receptor subtype [21]. It has been approved for the treatment of overactive bladder disorder since 2004. At recommended doses of 5 and 10 mg/day, peak plasma levels of solifenacin are attained within 3 to 8 h. Solifenacin is primarily metabolized in liver by the cytochrome P450 enzyme CYP3A4 and excreted by the kidneys with an elimination half-life of 45 to 68 h. It binds to hERG (human Ether-a-go-go-Related Gene) channels in the heart and may prolong the QT interval, although rarely to a clinically relevant degree [22]. Multiple preclinical and clinical studies indicate that solifenacin does not penetrate the blood–brain barrier sufficiently to have a meaningful effect on cognition at doses given in this trial [23].

## Patients and Methods

### Objectives and Study Design

The primary goal of this clinical trial was to determine the MTD of donepezil that could be safely given to patients with moderate AD when administered in combination with solifenacin as CPC-201. AS all those admitted to this study had been chronically treated with stable doses of donepezil for at least 3 months at their putative MTD of 10 mg/day, the donepezil dose increment enabled by the addition of a peripheral anticholinergic could be estimated. Secondarily, this study explored the potential effects of higher-dose donepezil to provide signals suggesting additional cognitive and global benefit.

In pursuit of this goal, a modified placebo-controlled, dose-escalation, crossover study was conducted at 7 sites in the USA from 19 August 2014 to 3 March 2016. The outpatient study used a modified single-blind design: patients and their caregivers, as well as cognitive measure raters and other study personnel, remained masked to treatment status, with the exception of the coordinator and a physician at each site who controlled dosing. Both the protocol and informed consent were approved by the independent ethics committee/institutional review board for each independent site and conformed to the principles of the World Medical Association Declaration of Helsinki and all local regulations. The study design was also reviewed and deemed appropriate by the US Food and Drug Administration (Investigational New Drug Application 114776).

### Study Population

Patients eligible for this trial ranged in age from 50 to 89 years and had a diagnosis of probable dementia of the Alzheimer’s type, as defined in the American Psychiatric Association’s Diagnostic and Statistical Manual of Mental Disorders [24], and based on National Institute of Neurological and Communicative Diseases and Stroke, Alzheimer’s Disease and Related Disorders Association criteria [25]; had a Mini-Mental State Examination (MMSE) score [26] of 10 to 20 (moderate impairment); were ambulatory and free of any medical condition that would adversely affect the safety or informative value of this study; and had clinical laboratory values within normal limits, or, if abnormal, considered by the investigator and sponsor to be not clinically significant.

All participants had been treated with donepezil 10 mg/day for at least 12 weeks prior to study entry. Patients taking memantine, at stable doses of either 20 mg/day immediate release or 28 mg/day extended release for at least 8 weeks before screening, were allowed to continue at that dose throughout the trial. All patients were required to have caregivers who either lived with or had daily contact with the patient and were not considered by the investigator to be cognitively impaired to a degree compromising their evaluations regarding safety/tolerability and global functioning.

Patients were excluded if they had a history or presence of seizures; myasthenia gravis; peptic ulcer; GI obstructive disorder or reduced GI motility; narrow-angle glaucoma; urinary retention; unexplained syncope; family history of sudden death; myocardial infarction or hospitalization for congestive heart failure within the prior 6 months; history of implanted cardiac pacemaker or implantable cardiac defibrillator; electrocardiogram (ECG) findings of prolonged QT interval, complete left bundle branch block, ventricular pacing, second-degree or third-degree atrioventricular block, atrial fibrillation or atrial flutter, heart rate <45 or >100 beats per min; PR > 220 ms, or QTcF >450 ms in males or >470 ms in females; patients treated with the following medications within 8 weeks of screening: AD treatments (other than donepezil and memantine), peripherally acting anticholinergics (e.g., drugs for the treatment of overactive bladder disorder), psychoactive medications (including antipsychotics, antidepressants, anxiolytics, or sedative hypnotics) having significant anticholinergic effects, and/or believed to affect alertness or cognitive function.

Prior to initiating study procedures, investigators obtained written informed consent from each patient, if possible, or from the patient’s legal authorized representative. If a patient was unable to provide written consent, he or she was required to provide verbal assent to participate in the study. Additionally, the caregiver or legal authorized representative was required separately to provide written informed consent for his or her own participation in the study.

### Study Drug Administration

The CPC-201 trial consisted of 4 phases (baseline, solifenacin titration, donepezil titration, and MTD maintenance), nominally lasting 2 days, 2 weeks, 12 weeks, and 12 weeks, respectively, for a total of approximately 26 weeks. Study medications were taken together, once daily in the morning throughout the trial. The blind was maintained by means of a double-dummy system. At every clinic visit patients received daily packs of 6 tablets; 2 similarly appearing tablets contained either active or placebo solifenacin, and 4 similarly appearing tablets contained either active or placebo donepezil.

Upon study admission, patients switched from their ongoing 10 mg/day dose of donepezil to the constant 6 tablet/day regimen. Initially, 1 donepezil tablet contained 10 mg, while all other tablets were inactive. Subsequently, in accordance with the protocol titration schedule, placebo solifenacin was replaced by active solifenacin at a dose of 10 mg/day and increased after 1 week to 15 mg/day. Donepezil was then increased by weekly 5-mg increments to 25 mg and thereafter at every-other-week intervals to each patient’s first intolerable dose or the protocol maximum of 40 mg/day. Patients attaining their first intolerable dose were immediately reduced to their previous MTD. Upon titration phase completion, all patients entered a MTD maintenance phase lasting 3 months. Study drug dosing was always permissive, consistent with patient needs and investigator discretion. Compliance, evaluated by counting unused tablets from each patient at every clinic visit, as well as by periodic measurements of peak and trough plasma levels of both study medications, was >98%. At the end of the study, all patients were given, at the investigators’ discretion, the option of entering a 6-month open-label extension or exiting after returning to their previous treatment regimen and receiving a final safety check.

### Safety and Efficacy Assessments

The primary outcome measure for this exploratory phase IIa study was the difference in the highest stably tolerated dose of donepezil when administered alone and when given together with solifenacin (15 mg/day). Secondary measures included safety–tolerability and initial efficacy assessments, conducted at regular intervals, as well as by standard laboratory tests. Patients returned to the clinic for evaluation at each dose adjustment or else at weekly intervals during dose titration (up to the week-14 visit) and at monthly intervals during MTD maintenance (at the week 18, 22, and 26 visits). Regular telephone contact between site staff and patient/caregivers maintained interim surveillance.

Safety assessments included clinical laboratory testing (hematology, biochemistry, and urinalysis panels analyzed by a central laboratory that met regulatory certification requirements), 12-lead ECG read centrally by a cardiologist with advanced training, and physical and neurologic examinations, including vital sign measurements, at all clinic visits. Blood pressure and heart rate were measured in the supine position. Temperature, respiratory rate, and weight were also determined at all clinic visits. Height was recorded at screening. In addition, possible deleterious effects of solifenacin on cognitive function were assessed by means of the Alzheimer’s Disease Assessment Scale Cognitive Component (ADAS-cog) scale at baseline and usually 2 weeks later when a dose of 15 mg/day had been achieved, both time points where the donepezil dose remained at 10 mg/day.

Treatment-emergent adverse event (TEAE) listings derived from spontaneous reports from patients and/or caregivers, as well as open-ended questioning, throughout the study. The severity of each TEAE (mild, moderate, or severe) and its relation to study medications (unrelated, possibly, probably, or definitely related) were determined by the investigators and reviewed by the Data Safety Monitoring Board (DSMB) and the sponsor.

Secondary outcome measures also included the 11-item ADAS-cog to evaluate safety, as well as a potential indicator of efficacy after completion of donepezil titration (at the week-12 visit) and again after 1, 2, and 3 months of MTD maintenance. In addition, the Clinical Global Impression of Improvement (CGI-I) scale was performed at study completion during the week-26 visit.

The ADAS-cog is an 11-item instrument commonly used in AD trials to assess cognitive function [27]. Disturbances in such core symptoms of AD as memory, language, praxis, and attention are evaluated. Total scores range from 0 (most impaired) to 70 (least impaired). Blinded, trained raters after fulfilling qualification standards and demonstrating satisfactory performance on tests of rating proficiency performed the ADAS-cog assessments. With few exceptions, patients were evaluated by the same rater throughout the trial.

The CGI-I is a semistructured tool that provides an overall assessment of how much a patient's illness has changed, whether or not entirely due to study medications, relative to their baseline state at the beginning of the intervention [28]. Patients were rated once at the end of the study independently by both the investigator and the caregiver on a 7-point scale (1 = marked improvement; 2 = moderate improvement; 3 = minimal improvement; 4 = no change; 5 = minimal worsening; 6 = moderate worsening; 7 = marked worsening). Raters were asked to rate overall change, taking into account all available information, compared with the patients’ condition at baseline [29]. For both the ADAS-cog and CGI-I measures, a negative change indicates improvement.

The MMSE is a 30-item test of cognitive function, with total scores ranging from 0 (most impaired) to 30 (least impaired) [26]. It was employed as a screening tool to determine admission eligibility. The same rater generally administered the ADAS-cog and then the MMSE.

The pharmacokinetics of donepezil alone (at baseline) and with solifenacin (at all subsequent times) were assessed at both trough (15 min before daily drug administration) and nominal peak drug levels (4 h later) at time points including baseline, end of solifenacin titration, end of donepezil titration, end of each month of MTD donepezil maintenance, and at study conclusion.

### Statistical Analysis

A sample size of 34 evaluable patients was planned to provide an overall power of ≥80% to observe a significant difference in the putative MTD of donepezil, prior to study entry when given alone at 10 mg/day and at the end of donepezil titration when given with solifenacin 15 mg/day as CPC-20.

Safety and tolerability were analyzed in the safety population (all those who received at least 1 dose of study medication). Efficacy analyses were performed on both the intention-to-treat (ITT) and the efficacy evaluable populations. The ITT was defined as all randomized patients who had at least 1 postbaseline assessment. The efficacy evaluable population was defined as all those who received study drugs, attended all study visits, and participated in all study procedures leading to the determinations. The primary efficacy endpoint (change in donepezil dose) was analyzed in the ITT population. The secondary efficacy measures (cognitive and global change) were assessed in the efficacy evaluable population. Post-hoc analyses of the impact of patient age, sex, baseline symptom severity, and concomitant medications on the response to treatment were conducted.

Since this initial phase II trial did not include a placebo arm, adjustment of ADAS-cog differences from baseline to changes from 10 mg donepezil and from placebo was based on an independently performed meta-analysis of the published literature [30]. Prior to breaking the blind, selection of randomized controlled trials for inclusion in this analysis relied on quality (internal validity) of search strategies and selection criteria developed by the Cochrane Collaboration [31] followed by comparability criteria devised a priori for this study, including such factors as disease severity, donepezil dose, treatment duration, and outcome measures [3, 32–37]. Similarly, the slope of cognitive decline during placebo or 10 mg/day donepezil treatment was estimated to be 0.0119 ADAS-cog points per day. Finally, a mean effect of 10 mg donepezil of 2.9 ADAS-cog points above placebo also derived from meta-analyses of data from multiple trials [31, 38–40].

The present study, the first phase II evaluation of CPC-201, was intended to be hypothesis raising rather than hypothesis testing; it was thus not powered to yield reliably statistically significant conclusions with respect to neurobehavioral efficacy. The data were analyzed by means of descriptive statistics (including means and SEs to reveal possible trends that might help guide the design of future, more definitive, clinical trials). An analysis of covariance model with terms for baseline score and treatment was used as the primary model for testing treatment effects on ADAS-cog scores ± SEM values used to compare treatment groups. For the categorical endpoint, CGI-I score at the 26-week visit a nonparametric analysis of covariance method combined with a Cochran–Mantel–Haenszel test component was used. All statistical tests, 2-sided at a significance level of 0.05, were conducted using SAS version 8.0 or higher [41] by Amarex LLC [30]. An independent safety monitoring board (DSMB) determined that the study could proceed as planned.

## Results

### Patient Baseline Characteristics and Disposition

Baseline demographic and clinical characteristics of the 41 patients comprising the ITT/safety population are presented in Table 1. Eligible patients ranged in age from 57 to 88 years. Most were white (93%) women (54%). MMSE scores fell in the moderate range of 10 to 20; 61% continued to receive their established dose of memantine throughout the trial. Other frequent concomitant medication classes used during the study included lipid-lowering agents (58%), antidepressants (54%), and antihypertensives (44%).

**Table 1:** Summary of baseline demographics of Alzheimer’s disease patients entering CPC-201 trial

Characteristic	*n* (%)
Sex	
Male	19 (46.3)
Female	22 (53.7)
Mean ± SD age (y)	73.1 (8.2)
Age range (y)	57–88
Race	
White	38 (92.7)
Black/African American	3 (7.3)
Asian	0 (0)
Mean ± SD weight (kg)	76.4 ± 18.0
Weight range (kg)	40–117
Mean ± SD (median) duration of Alzheimer’s disease (y)	3.4 ± 2.7 (3.0)
Range	0.5–11
Mean ± SD duration of donepezil treatment prior to study entry (d)	833.8 ± 718.3
Range	70–2680
Concomitant memantine	
Yes	25 (61.0)
No	16 (39.0)
Mean ± SD baseline ADAS-cog	28.5 ± 8.3
Range	15–46
Mean ± SD baseline MMSE	16.5 ± 3.10
Range	11–20

Data are *n* (%) unless otherwise indicated

ADAS-cog = Alzheimer’s Disease Assessment Scale Cognitive Component; MMSE = Mini-Mental State Examination

**Fig. 1:**
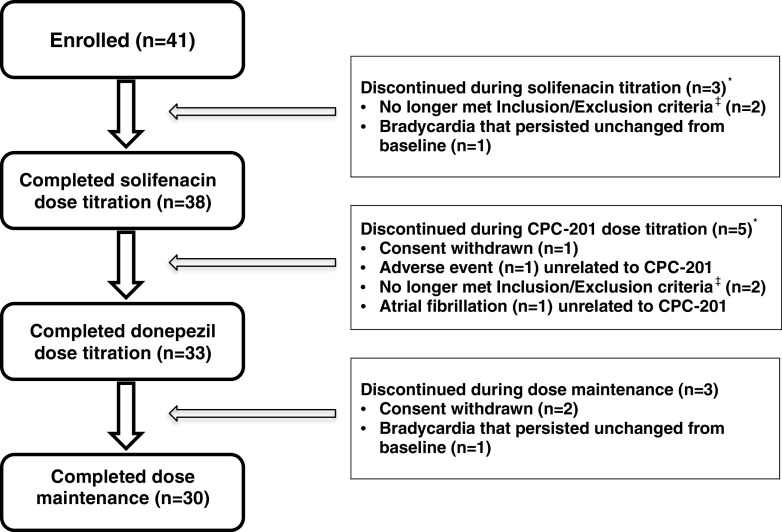
Disposition of patients with moderate Alzheimer’s disease enrolled in the study of CPC-201. No patient discontinued owing to possible or probable drug-related adverse events or to a perceived lack of efficacy. *Of 8 patients who discontinued during titration, 3 occurred during solifenacin titration and 5 during donepezil titration ^‡^Post-enrollment, 4 patients were excluded as ineligible pursuant to protocol

Figure 1 summarizes the disposition of the ITT population. Eleven premature withdrawals occurred during this trial: 8 patients dropped out during initial solifenacin or donepezil titration, and 3 during stable dose maintenance. The reasons were: nonconformance with inclusion/exclusion criteria (4 patients), consent withdrawal (3 patients), bilateral plantar dermatitis unrelated to study drugs (1 patients), bradycardia unrelated to study drugs that persisted unchanged from baseline (2 patients), and atrial fibrillation unrelated to study drugs discovered at an in-clinic visit during donepezil upward dose titration (1 patient). No withdrawal was attributed by the investigator or the DSMB to a drug-related AE.

### Solifenacin Administration

Solifenacin was given orally at a daily dose of 10 mg for 1 week and then increased to 15 mg for the remainder of the trial. The peripheral anticholinergic produced no untoward clinical or laboratory effects in the 41-patient safety population. Specifically, there were no symptoms of neuropsychological dysfunction reported, and cognition measured by the ADAS-cog after 2 weeks of solifenacin treatment did not change [mean ± SEM of 26.9 ± 1.25 at baseline (donepezil 10 mg/day only) *vs* 26.9 ± 1.28 after treatment (donepezil 10 mg/day plus solifenacin 15 mg/day) for a difference of 0.012 ± 0.76 (*n* = 26; *p* = nonsignificant)]. At no later time during the study were centrally mediated untoward effects of solifenacin detected or a need for a downward dose adjustment recognized.

### Primary Endpoint

#### Maximum Tolerated Dose of Donepezil

Patients admitted to this study had been treated with standard donepezil (10 mg/day) for at least 3 months. Clinically, all were considered by their investigator to be responding satisfactorily. Once a solifenacin dose of 15 mg/day had been achieved, the amount of donepezil was gradually increased to the protocol maximum of 40 mg/day or to each patient’s first intolerable dose (then reduced to the next lower dose). Donepezil titration took an average of 9.6 ± 0.62 weeks, prolonged by efforts to accommodate to such matters such as scheduling convenience, as well as drug tolerability.

**Fig. 2:**
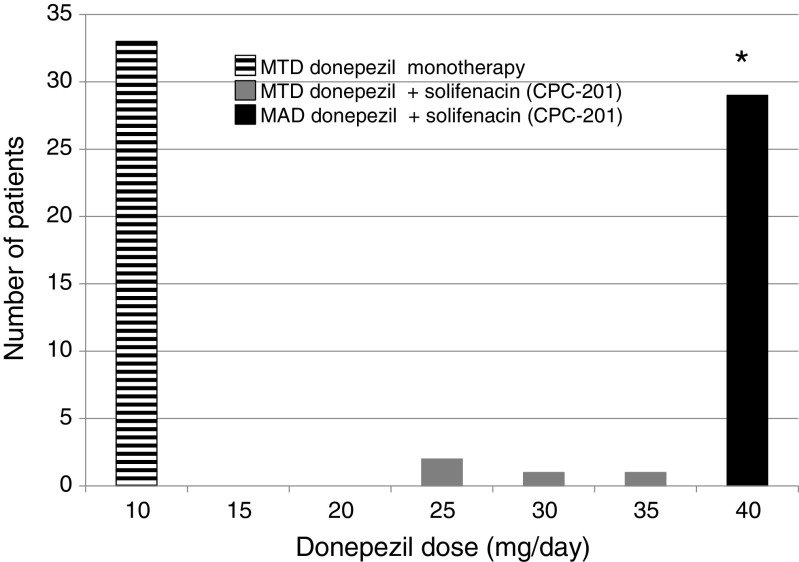
Maximum tolerable dose (MTD) of donepezil during preadmission monotherapy (10 mg/day) and MTD or maximum allowed donepezil dose (MAD = 40 mg/day) at completion of donepezil plus solifenacin (CPC-201) dose titration. Histogram compares donepezil doses in 33 patients with moderate AD, as determined first by history (from investigators or referring physicians) and again at completion of donepezil (plus solifenacin 15 mg/day) dose titration. A maximum donepezil dose of 25 mg/day was tolerated by 100% and of 40 mg/day by 88% of CPC-201 treated patients (*p < 0.001 for difference in donepezil dose administered as monothrerapy at baseline and co-administered with solifenacin as CPC-201 at the end of dose titration)

All patients completing donepezil dose titration attained the primary goal of safely and tolerably increasing their MTD of this ChEI when co-administered with the anticholinergic solifenacin (Fig. 2). Indeed, 100% reached 25 mg/day and 88% of the 33 evaluable patients tolerated the maximum protocol allowable dose of 40 mg/day. The mean donepezil dose in this group was 38 ± 0.74 mg/day (*p* < 0.0001) and the median dose was 40 mg/day or 4-fold above that tolerated prior to admission. This dose increment was stably maintained throughout the final 3 months of the study. Three of the 4 (12%) patients who failed to attain a donepezil MTD of at least 40 mg/day evidenced GI intolerance and plasma donepezil levels approximating those occurring with a 40 mg/day dose in this total study population.

### Secondary Endpoints

This phase IIa study mainly conducted to determine the size of its primary efficacy outcome measure. It was neither designed nor powered to provide statistical assessments of secondary measures of safety or efficacy, but rather to suggest data trends suitable for possible future evaluation.

#### Safety—Tolerability

TEAEs, whether or not considered CPC-201 related, that occurred in more than 1 individual are presented in Table 2. Most emerged during the period of donepezil dose titration (71% of patients), less during donepezil MTD maintenance (58%), and least during the solifenacin titration phase (12%). The most frequently reported TEAEs deemed probably related to CPC-201 treatment involved the GI system. These occurred mainly during donepezil dose titration and included diarrhea (6 of 38 patients; 16%), nausea (4 of 38; 11%), and vomiting (4 of 38; 11%). In contrast, during CPC-201 MTD maintenance, only 2 patients (6%) developed diarrhea, and none had nausea or vomiting. Regarding solifenacin, just 1 individual (3%) was considered to have a TEAE (constipation) possibly or probably related to the anticholinergic during donepezil MTD maintenance.

**Table 2:** Treatment emergent adverse events (TEAEs) occurring in more than 1 patient, by treatment period, and preferred term, by descending order of frequency during donepezil titration period

TEAE preferred term		Number (%) of patients					
		Solifenacin upward dose titration		Donepezil upward dose titration		Donepezil dose maintenance	
		(*n* = 41)		(*n* = 38)		(*n* = 33)	
		*n*	(%)	*n*	(%)	*n*	(%)
Number of patients with any TEAE		5	(12.2)	27	(71.1)	19	(57.6)
	Diarrhea			6	(15.8)	2	(6.1)
	Nausea			4	(10.5)		
	Vomiting			4	(10.5)		
	Abdominal discomfort			3	(7.9)	1	(3.0)
	Decreased appetite			3	(7.9)		
	Dizziness			3	(7.9)	2	(6.1)
	Constipation	1	(2.4)	2	(5.3)	1	(3.0)
	Agitation			2	(5.3)	1	(3.0)
	Somnolence			2	(5.3)		
	Syncope			2	(5.3)	1	(3.0)
	Tremor			2	(5.3)	1	(3.0)
	Pyrexia			2	(5.3)		
	Fall	1	(2.4)	1	(2.6)	4	(12.1)
	Abdominal pain	1	(2.4)	1	(2.6)	1	(3.0)
	Bradycardia	1	(2.4)	-	-	1	(3.0)
	Electrocardiogram QT prolonged			1	(2.6)	1	(3.0)
	Arthralgia					2	(6.1)
	Paresthesia			1	(2.6)	1	(3.0)
	Irritability			1	(2.6)	1	(3.0)

Most TEAEs, drug related or not, were mild (68% of patients) or moderate (54%) in severity. During this trial, severe AEs occurred in 7 (17%) patients. All were unique and none was considered related to treatment. Serious TEAEs occurred in 8 patients (20%); all were considered unrelated to treatment. Only 1 patient prematurely withdrew from the study due to a TEAE: bilateral plantar dermatitis judged unrelated to CPC-201 treatment. The death of 1 patient (from pneumonia 2 weeks after stopping study medications) was also not considered related to the treatment.

During the entirety of this trial, there were no clinically meaningful changes in clinical, vital signs (blood pressure, heart rate, or heart rhythm), or standard laboratory assessments. Although no significant change in mean body weight occurred, decreased weight as an AE was reported in 1 patient (2%). Compared with baseline, 2 patients (5%) had a weight decrease of ≥7% at the end of the study. QTcF intervals, read centrally from routine paper ECGs obtained 4 h postdrug intake and at each dose increase and at monthly intervals during maintenance, averaged (mean ± SD) 421 ± 22.2 ms at baseline (10 mg/day donepezil monotherapy; *n* = 41), 436 ± 23.7 ms at solifenacin steady state (*n* = 35), and 436 ± 22.6 ms at the end of the 12-week maintenance (*n* = 30). There was no correlation between QTcF change and plasma donepezil or solifenacin concentrations and all QTcF increments remained <60 ms from baseline, except for 1 individual who had brief, transient increases to 78 ms.

#### Cognitive Function

**Fig. 3:**
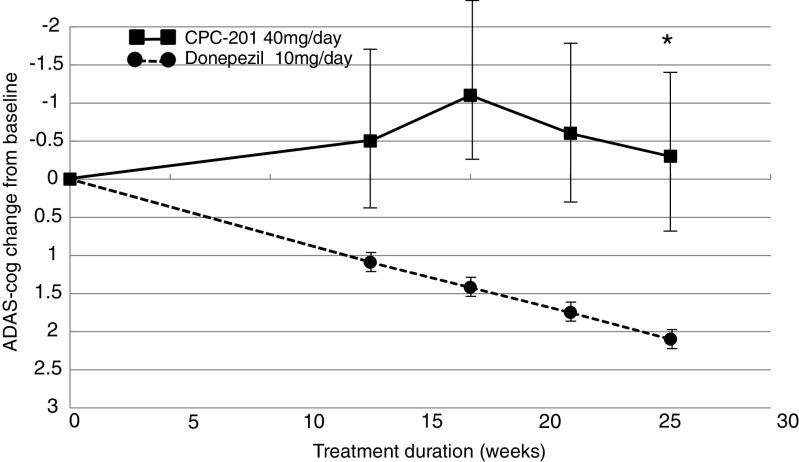
Time course of Alzheimer’s Disease Assessment Scale Cognitive Component (ADAS-cog) score (mean ± SEM) response to donepezil 40 mg/day plus solifenacin 15 mg/day (CPC-201) in 23–26 efficacy evaluable patients with moderate Alzheimer’s disease during the 26-week study and to donepezil 10 mg/day monotherapy as derived from meta-analysis of comparable randomized controlled trials as described in text. Donepezil dose titration until week 12 was followed by stable dose maintenance for 14 additional weeks. Negative ADAS-cog values indicate improvement *p = 0.051 for difference from donepezil monotherapy

ADAS-cog scores showed a positive treatment effect that remained above baseline throughout the course of this 26-week study (Fig. 3). Cognitive improvement peaked following completion of donepezil titration to MTD at the 18-week visit. Subsequently, mean ADAS-cog scores declined at an ostensibly linear rate that appeared to parallel the historic slope of 10 mg/day donepezil monotherapy, estimated as described in the “Patients and Methods” section. During the stable-dose maintenance period, cognitive benefit from CPC-201 averaged about 2.5 points above that estimated for standard 10 mg/day donepezil (*n* = 23, *p* < 0.05; Table 3). Further adjustment of this ADAS-cog benefit for that already received from the 10 mg/day dose of donepezil all patients were receiving at study entry (based on the previously described meta-analysis) suggested that CPC-201 produced a mean improvement of 5.4 ± 0.84 points over placebo at trial completion.

**Table 3:** Effect of CPC-201 treatment on cognitive function in patients with moderate Alzheimer’s disease measured by Alzheimer’s Disease Assessment Scale Cognitive Component (ADAS-cog) changes from baseline and estimated from 10 mg donepezil and from placebo response

Mean treatment weeks	Median donepezil dose (mg/kg)	Number of patients	ADAS-cog difference from:		
			Baseline*	Aricept 10 mg^†^	Placebo^†^
14	40	26	−0.55 ± 0.92	−1.6 ± 0.88	−4.5 ± 0.88^‡^
18	40	25	−1.1 ± 1.1	−2.5 ± 1.1^‡^	−5.4 ± 1.1^‡^
22	40	24	−0.61 ± 1.1	−2.4 ± 1.1^‡^	−5.3 ± 1.1^‡^
26	40	23	−0.35 ± 0.85	−2.5 ± 0.84^‡^	−5.4 ± 0.84^‡^

ADAS-cog scores from the evaluable population are the means ± SEM, with negative scores indicating improvement

*Differences from baseline measured during stable maximum tolerated dose maintenance at the times after CPC-201 treatment initiation specified

^†^Differences from values for 10 mg/day donepezil and placebo derive from meta-analyses of results from comparable randomized controlled trials meeting Cochrane Collaboration quality guidelines, as described in the text

^‡^*p* < 0.05

Responder analysis of the completed trial results showed that 14 (61%) of the efficacy evaluable population had ADAS-cog change scores of 0 to −7 and were thus regarded as CPC-201 responders, while 9 (39%) had change scores <0 and were considered nonresponders. At the end of the 26-week trial, ADAS-cog increments for responders averaged −2.9 ± 0.77 points above baseline and −7.9 ± 0.77 points above placebo (both *p* < 0.05 for *n* =14). Indeed, all 14 of the responding individuals had estimated ADAS-cog benefit above placebo of at least 4 points.

Domain analysis of the ADAS-cog results at trial conclusion revealed that Memory [sum of items 4 (Word recall), 6 (Orientation), and 10 (Word recognition)] responded substantially better than Language [Sum of Item 1 (spoken language ability), Item 2 (Comprehension), Item 3 (Word finding difficulty), Item 5 (Naming objects and fingers), and Item 11 (Remembering test instructions)] or Praxis [Sum of Items 7 (commands), 8 (ideational praxis), and 9 (constructional praxis)]. Moreover, mean baseline scores for the 3 items comprising the memory domain averaged substantially worse (7.01) than those for the remaining 8 ADAS-cog items (0.85). The severity of memory dysfunction thus might serve as a possible predictor of the response to strong cholinomimetic stimulation.

#### Global Function

**Fig. 4:**
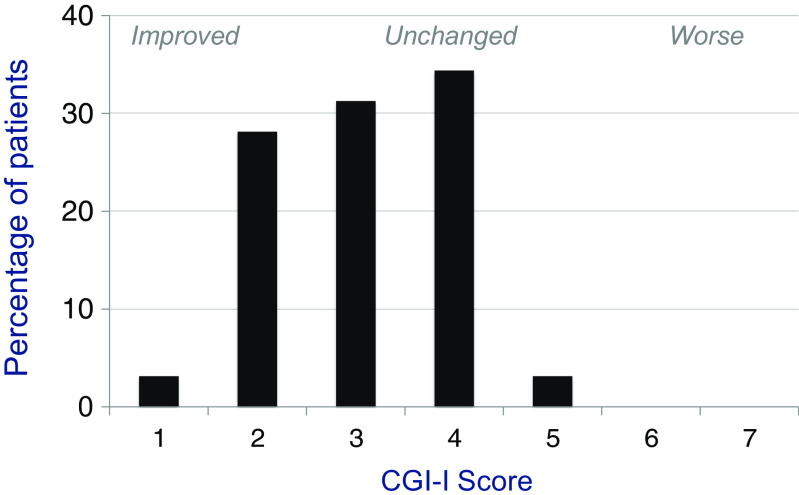
Histogram of global response to donepezil (median dose of 40 mg/day) plus solifenacin (15 mg/day) administered as CPC-201 at end of 26-week study in 11 efficacy evaluable patients with moderate Alzheimer’s disease. The Clinical Global Impression of Improvement (CGI-I) was scored on a 7-point scale by both investigators and caregivers

The CGI-I results indicated substantial global improvement at the end of this 26-week trial (Table 4). Scores obtained independently from investigators and caregivers from all those in the efficacy evaluable population receiving this test did not differ significantly but averaged somewhat higher from caregiver group. Independently and in combination CGI scores revealed significant benefit. At study conclusion, investigator, caregiver and combined CGI score all improved significantly from the pretreatment baseline (*p* < 0.001), the latter by an average of 0.94 ± 0.20 points (*n* = 16 in efficacy evaluable population). Responder analysis indicated that all but 1 individual in this group were considered to have improved with CPC-201 therapy (Fig. 4).

**Table 4:** Effect of 26 weeks of CPC-201 treatment on global function in patients with moderate Alzheimer’s disease as measured by the Clinical Global Impression of Improvement (CGI-I) scale

Rater	CGI-I score (mean ± SEM)	Change from baseline (mean ± SEM)
Investigator	3.3 ± 0.19	−0.75 ± 0.19*
Caregiver	2.9 ± 0.27	−1.1 ± 0.27*
Combined	3.1 ± 0.20	−0.94 ± 0.20*

Values are from 16 evaluable patients at the completion of 26 weeks treatment with CPC-201 containing a median donepezil dose of 40 mg/day. Baseline score is 4 (no change) on a 7-point scale ranging from 1 (marked improvement) to 7 (marked worsening). Negative changes indicate improvement

*p < 0.01

#### Predictors of Treatment Response

None of the demographic or other patient characteristics measured at baseline in this study were found on post hoc analysis to relate significantly to changes in overall cognitive or global function. More specifically, neither age, sex, baseline dementia severity, nor concomitant memantine appeared to affect the CPC-201 response as measured by the ADAS-cog or CGI-I in this small patient sample. However, patients continuing to receive their previous dose of memantine tended to have a larger ADAS-cog response above baseline than those not receiving memantine, although this difference did not attain statistical significance (*p* > 0.05).

#### Pharmacokinetics

**Fig. 5:**
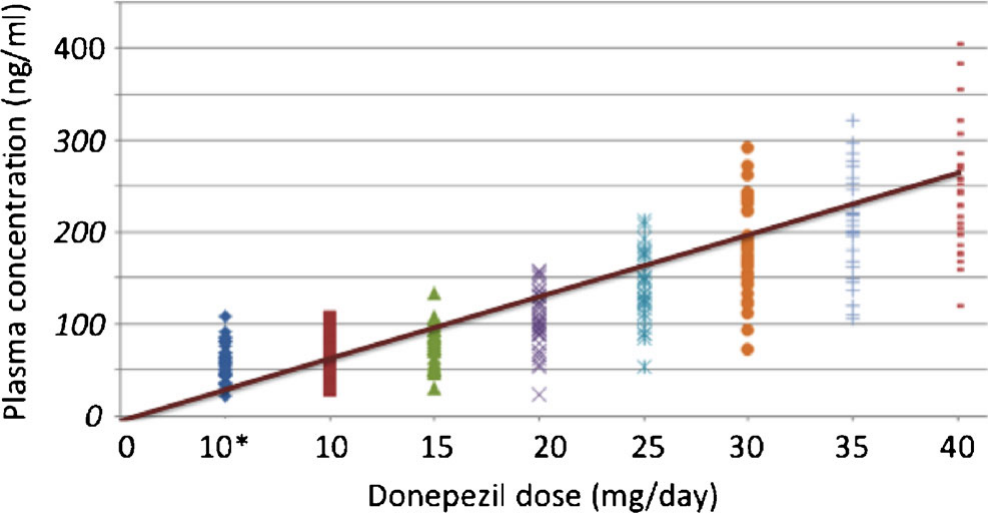
Peak plasma donepezil (C_max_) concentrations at all doses administered during ascending CPC-201 dose titration to 41 patients with moderate Alzheimer’s disease. *No solifenacin co-administration; all other donepezil doses given with 15 mg/day solifenacin

Mean peak plasma donepezil concentrations increased linearly over the range of ChEI doses studied (Fig. 5). Donepezil levels averaged 57 ± 3.4 ng/ml (*n* = 41) at baseline (10 mg/day donepezil monotherapy) and 67 ± 4.0 ng/ml after the addition of 15 mg/day solifenacin (*n* = 36; *p* > 0.05 for difference from donepezil alone). Following titration to a median dose of 40 mg/day (in combination with solifenacin), mean donepezil concentrations increased to 258 ± 14.2 ng/ml (*n* = 32), ranging from 104 to 420 ng/ml. This 4.5-fold increase in plasma donepezil remained essentially stable throughout the dose maintenance phase of the study. Solifenacin levels averaged 64 ± 4.6 ng/ml (*n* = 37) at the end of the 2-week titration to 15 mg/day and 78 ± 7.2 ng/ml (range 24–194 ng/ml; *n* = 29) at study conclusion. No significant drug–drug interactions or drug–efficacy correlations emerged from examination of this relatively limited data set.

## Discussion

This small, first-in-patient clinical trial found that solifenacin co-administration enabled the dose of donepezil to be safely increased to 40 mg/day in 88% of patients with moderate AD. Treatment with high-dose CPC-201, which combines the ChEI with the peripheral anticholinergic, resulted in a more than 4-fold increment in circulating donepezil concentrations and thus brain exposure to the therapeutic agent. Solifenacin administration, in amounts that failed to produce any detrimental effects on cognition, allowed this dose increase by diminishing all TEAEs of donepezil, especially those involving the GI system. At trial conclusion, measures of both cognitive and global function suggested significant improvement with high-dose donepezil-containing CPC-201 over standard 10 mg donepezil in patients with moderate AD.

The present results with solifenacin co-administration support the hypothesis that the dose-limiting AEs of donepezil-like ChEIs reflect peripheral, not central, muscarinic receptor stimulation [19]. They are also consistent with the view that profound under dosage contributes to the meager antidementia efficacy of currently approved donepezil treatments [7, 10, 19]. Finally, the findings of this study add strength to the view that higher ChEI doses, within the range evaluated here, may bring greater cognitive benefit to patients with AD [3, 10–18].

All 33 efficacy evaluable patients completing the drug titration phase tolerated CPC-201 containing at least 25 mg/day of donepezil (thus exceeding the highest currently approved donepezil dose) and nearly all (88%) reached the per-protocol maximal allowable dose of 40 mg/day. Plasma donepezil concentrations in the 4 (12%) individuals who did not attain MTDs of at least 40 mg/day averaged well above those expected from the dose they received. An ongoing extension study suggests that donepezil doses up to at least 60 mg/day (combined with solifenacin 15 or 20 mg/day) are well tolerated by most patients with AD. By contrast, repeated doses of donepezil monotherapy in the 45 to 180 mg/day range (plasma concentrations extending from 55 to 546 ng/ml) are reported only in the toxicology literature, usually in association with unintentional overdose and severe adverse effects [42, 43]. Pharmacokinetic results, notwithstanding relatively wide scatter, confirm that higher donepezil doses bring proportionally greater plasma levels.

No medically significant safety issues emerged during the course of this trial. High donepezil doses failed to slow significantly heart rate or reduce blood pressure. The well-known tendency of solifenacin to prolong the QT interval was observed [22], but when co-administered with 40 mg/day donepezil this effect failed to worsen to a clinically significant degree. Similarly, no deleterious cognitive consequences attended solifenacin administration.

AE frequency during the high-dose donepezil containing CPC-201 maintenance phase decreased by about 80% of that observed in comparable randomized control trials of 10 mg/day donepezil [44]. High-dose CPC-201 appeared especially superior to currently available donepezil dosage forms in relation to GI tolerance. Indeed, the most frequent AEs during CPC-201 maintenance were injuries due to accidental falls in 4 individuals, 3 of whom had a history of frequent falls. The dearth of solifenacin-related AEs in this study suggests that the cholinomimetic and anticholinergic components of CPC-201 tend to mutually antagonize the peripherally mediated effects of the other.

This exploratory trial was neither designed nor powered to detect reliably improvement in secondary behavioral outcomes measures such as the ADAS-cog or CGI-I. These scales were primarily intended to detect possible centrally mediated adverse effects of high-dose solifenacin. Nevertheless, ADAS-cog scores remained consistently above baseline throughout the administration of high-dose CPC-201. At study end, both the ADAS cog and CGI-I results were significantly improved compared with their baseline values (*p* < 0.05). Indeed, the estimated 5.4-point ADAS-cog increment over placebo, as well as the 0.94-point CGI-I benefit above baseline, appear to exceed that reported for any currently approved AD medication [10, 31, 38–40]. For the 61% of the efficacy evaluable patients considered to be CPC-201 responders, all enjoyed a clinically meaningful cognitive improvement of at least 4 ADAS-cog points.

Some animal model studies suggest that the response to ChEIs such as donepezil assumes an inverted U-shaped dose response at high doses [45]. While true at excessive (barely subtoxic) amounts [46], the present results appear to provide no support for this view in patients with AD receiving up to 40 mg/day of donepezil. Indeed, comparison of 40 mg/day donepezil-containing CPC-201 results with those for 5, 10, and 23 mg/day suggest an essentially dose-proportional increase in the range studied [10, 31]. The ADAS-cog response to the 40 mg/day donepezil-containing CPC-201 appeared nearly double (occurring with the standard 10 mg/day donepezil formulation) [31, 38–40]. These preliminary observations thus lend support to the view that donepezil doses above those currently approved may actually confer greater antidementia efficacy.

A diagnosis of AD was required for inclusion in this trial. However, a possibly better target for cholinomimetic interventions like CPC-201 might be those suffering from a hypocholinergic dementia, whether or not they satisfy AD diagnostic criteria. Unfortunately, as yet, there is no clinical means to identify reliably those with a cognitively significant loss of cerebral cholinergic transmission. In the present study, individuals with the greatest deficit in ADAS-cog items making up the memory domain tended to be those having the greatest memory item and overall cognitive response to CPC-201 [47]. Going forward, it may be important to find dependable ways to identify optimal ChEI responders to better focus therapeutic interventions and improve the cost-effectiveness of symptomatic antidementia therapies [48].

Both the degree of improvement in CGI-I scores and the proportion of patients improving appeared larger than reported from earlier trials of 10 mg/day donepezil using the similar CIBIC+ scale [32, 33, 35]. Indeed, the 23-mg donepezil study in patients with severe AD reported no apparent global improvement between 10 and 23 mg doses [10]. In contrast, all but a single individual in the present trial were judged unchanged or improved on the CGI-I scale. Conceivably, there may be neurobehavioral domains benefitted by CPC-201 not captured on the ADAS-cog, since, as commonly reported, only about 60% of patients responded to this scale [49]. Assuming only those having a cholinergic deficit that substantially contributes to their dementia will significantly benefit from cholinomimetic therapy, the present results could suggest that brain hypocholinergia contributes to the memory loss occurring in almost two-thirds of patients diagnosed with mid-stage AD and especially to the various associated disturbances (as assessed by broader CGI type psychometric measures) that manifest in a considerably larger proportion of these individuals.

Conclusions regarding a potentially improved efficacy of high-dose donepezil in CPC-201 in those with mid-stage AD based on the findings of the present exploratory study must be regarded with caution. Nevertheless, the results encourage further evaluation of this possibility in a fully powered, randomized, and controlled clinical trial.

## Electronic supplementary material

Below is the link to the electronic supplementary material.

ESM 1 (PDF 523 kb)
